# A Case of Primary Lymphoepithelioma-Like Carcinoma of the Bladder With Review

**DOI:** 10.7759/cureus.40433

**Published:** 2023-06-14

**Authors:** Gangfu Zheng, Nengfeng Yu, Jiaqi Du, Yichun Zheng

**Affiliations:** 1 Department of Urology, The Fourth Affiliated Hospital, International Institutes of Medicine, Zhejiang University School of Medicine, Yiwu, CHN

**Keywords:** bladder mass, laparoscopic partial cystectomy, surgical case reports, urinary bladder carcinoma, lymphoepithelioma-like carcinoma

## Abstract

Lymphoepithelioma-like carcinoma (LELC) was characterized by epithelial neoplastic cells developing in solid or incohesive sheets mixed with a noticeable lymphoid infiltration. Lymphoepithelioma-like carcinoma of the bladder (LELCB), which was first described by Zukerberg, is a rare variant of LELC. Here we reported a new case of LELCB occurring in a 70-year-old woman presenting with hematuria. Computed tomography (CT) and cystoscopy revealed a tumor on the left upper wall of the bladder. A partial cystectomy was finally performed. Pathological and immunohistochemical analysis revealed LELCB. After receiving systemic adjuvant chemotherapy, the patient conducted a 25-month follow-up without experiencing a recurrence.

## Introduction

Lymphoepithelioma-like carcinoma (LELC) or lymphoepithelioma carcinoma is a rare malignant epithelial tumor characterized by lymphocytic interstitial hyperplasia [1]. It is most common in the nasopharynx [2], followed by the esophagus [3], thymus [4], lungs [5], stomach [6], and liver [7]. Primary LELC of the urinary system is rare. Zukerberg et al. described primary LELC of the bladder (LELCB) for the first time in 1991 [8]. This study reported one case of primary LELCB and conducted a preliminary discussion on its clinical features, pathological morphology, clinical treatment, and other conditions based on previous LELCB case reports.

## Case presentation

**Figure 1:**
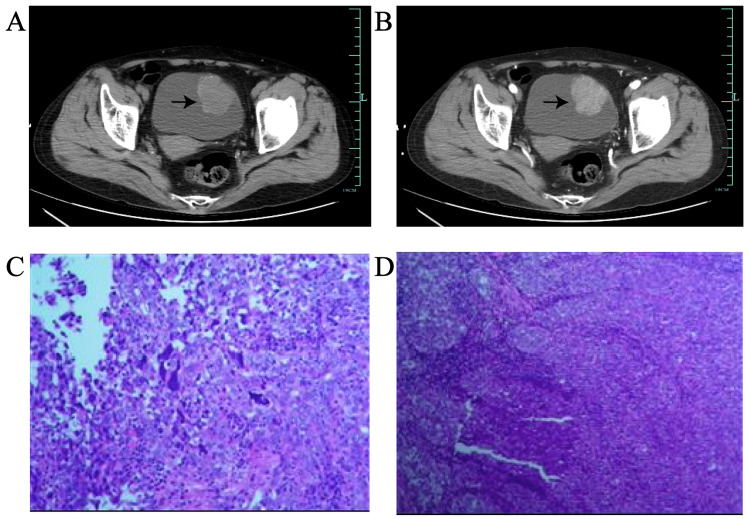
(A-B) Scanographic aspect of the bladder mass (arrowhead). (C-D) Microscopic aspect of lymphoepithelial-like carcinoma of the bladder.

A 70-year-old woman presented to our hospital in December 2020, complaining of intermittent painless gross hematuria for 20 days. She had no prior personal or family medical history of this complaint and also denied any family history of tumors or genetic diseases. In terms of patient history, the patient had a history of hypertension and the blood pressure could be controlled well. There were no notable findings in blood tests but urine cytology results were positive. And she didn't in an immunocompromised, HIV, or impaired glucose tolerance status. Computed tomography (CT) scan of the entire abdomen showed a cauliflower-like soft tissue mass on the left anterior wall of the bladder, with a size of approximately 3.6 x 4.2cm. Calcification could be seen at the edge (Figure 1A). And the enhanced CT scan showed significant enhancement (Figure 1B). On cystoscopy, one bladder tumor was observed on the left upper wall of the bladder, whose range was approximately 4cm. A total body CT scan was performed, and the presence of distant metastases or lymph node involvement was excluded. As a result, the clinical stage was diagnosed as T_2_N_0_M_0_ based on the UICC classification.

A transurethral resection of a bladder tumor (TURBT) was performed on December 11, 2020. Pathological findings revealed carcinoma cells with obvious nuclear atypia ranging in a nest-like, invasive growth and the infiltration of lymphocytes. The final pathological diagnosis was LELC with poor differentiation (Figure 1C and Figure 1D). These results indicated a muscle-invasive pT2 poorly differentiated carcinoma of the urinary bladder. Immunohistochemical results were as follows: EBER (-), P63(-), CK(AE1/AE3) (+), CK (34βE12) (+), CK20(-), CK7(+), EMA (+) and GATA3(-). The patient underwent adjuvant chemotherapy with gemcitabine and cisplatin one month after surgery and the chemotherapy plan was as follows: 1200mg of gemcitabine intravenously on days 1 and 9, and 80mg of cisplatin intravenously on day 2. However, the result of abdominal enhanced CT three months after surgery indicated cancer recurrence. Laparoscopic combined ureteroscopic partial cystectomy was then performed on March 31, 2021. Pathological examination revealed patchy diffuse infiltration of tumor cells in the bladder muscle layer and surrounding adipose tissue, with a large amount of lymphoid tissue in the stroma, which was also consistent with LELCB. As adjuvant chemotherapy, cisplatin and gemcitabine-based systemic chemotherapy were performed and it was repeated every four weeks, a total of three times. The plan was as follows: 1000mg of gemcitabine intravenously on days 1 and 8, and 35mg of cisplatin intravenously on days 2, 3, and 4. What’s more, she was treated with a regular bladder infusion of 1000mg gemcitabine every week, a total of 17 times. Cancer recurrence was not apparent at the usual follow-up as determined by CT scan and cystoscopy. The patient remained alive and free of cancer 25 months postoperatively.

## Discussion

An undifferentiated nasopharyngeal carcinoma, which was characterized by epithelial neoplastic cells developing in solid or incohesive sheets mixed with a noticeable lymphoid infiltration, was first named lymphoepithelioma by Carbone in 1982 [9]. LELC had similar histological features with lymphoepithelioma but primarily occurred in other organs, such as the esophagus [3], thymus [4], lungs [5], stomach [6], and liver [7]. LELC in the urinary system is rare, most common in the bladder, followed by the renal pelvis [10], ureter [11], and urethra [12].

Primary LELC of the urinary system is rare. Zukerberg et al. described primary LELCB for the first time in 1991 [8]. It was reported that the incidence of LELCB in all bladder cancer was 0.3-1.3% [13]. Intermittent painless gross hematuria and bladder lumps found on physical examination were the main reasons why patients often sought medical attention. Most of them were in the T2-T3 stage though [8,13]. Some studies found that LELC in the upper respiratory tract such as the nasopharynx and salivary glands was closely related to EB virus infection [14]. However, the role the EB virus played in LELCB remained unclear yet. It was reported that the high expression of p53 might be related to the occurrence and development of LELCB [15]. In general, the mechanism by which LELCB occurs and develops is still not sure.

In the WHO (2016) classification of tumors of the urinary system, LELCB had been separately listed as a special subtype of urothelium tumor and described in detail [16]. LELCB cells exhibited a syncytial appearance, with cytoplasm fused and unclear boundaries. The cytoplasm was lightly stained or slightly eosinophilic, with a large pleomorphic nucleus and prominent nucleoli. The intercellular space of tumor cells was mainly composed of lymphoid stroma, which contained T cells, B cells, and plasma cells [16,17]. What is more, according to the proportion of LELC and other neoplasms including urothelial carcinoma and squamous cell carcinoma, Amin et al. classified LELCB as pure (100%), prominent (>50%), and localized (50%). Patients with pure and predominant LELCB were more likely to have a better prognosis [18].

Immunohistochemistry (IHC) staining was universally acknowledged as a method for distinguishing LELCB from other types of bladder cancers. It also could differentiate malignant epithelial cells of LELC from normal inflammatory cells. The general results of IHC of LELCB were p53(+), EMA (+), CK3(+), CK20(+), lymphocyte markers CD3(+), CD20(+), CD138(+), NSE (-), STN (-), LCA (-), S-100(-) [19], which were almost consistent with the IHC results of this case.

Currently, there is no standard treatment plan for LELCB yet. Therefore, we have sorted out some optional treatments based on existing reports. For primary therapies, there are currently available therapeutic procedures such as TURBT, partial cystectomy, and radical cystectomy, while for adjuvant treatments there are systemic chemotherapy, irradiation, combined chemotherapy and radiotherapy, and intravesical chemotherapy. Collecting data from 56 patients, Serrano et al. came to the conclusion that patients with pure or predominant tumors could be treated with bladder-preserving treatments, whereas focal LELCB was more aggressive and necessitated cystectomy. Numerous chemotherapy regimens have been used with encouraging outcomes, including MVAC (methotrexate, vinblastine, doxorubicin, and cisplatin) and GC (gemcitabine and cisplatin) [13]. In order to have a more accurate understanding and use more effective treatments for this uncommon disease, more researches and managements are required.

## Conclusions

Here we reported a new clinical case of LELCB who was treated with partial cystectomy and adjacent chemotherapy and conducted a 25-month follow-up without experiencing a recurrence. Currently, there is no standard treatment plan for LELCB. For primary therapies, there are currently available therapeutic procedures such as TURBT, partial cystectomy, and radical cystectomy, while for adjuvant treatments there are systemic chemotherapy, irradiation, combined chemotherapy and radiotherapy, and intravesical chemotherapy. More researches and managements are required in the future.
